# Sensation of the autologous reconstructed breast improves quality of life: a pilot study

**DOI:** 10.1007/s10549-017-4547-3

**Published:** 2017-10-25

**Authors:** Anouk J. M. Cornelissen, Jop Beugels, Sander M. J. van Kuijk, Esther M. Heuts, Shai M. Rozen, Aldona J. Spiegel, René R. W. J. van der Hulst, Stefania M. H. Tuinder

**Affiliations:** 1 Department of Plastic Surgery, Maastricht University Medical Center +, P. Debyelaan 25, 6229 HX Maastricht, The Netherlands; 2 Department of Clinical Epidemiology and Medical Technology Assessment, Maastricht University Medical Center +, P. Debyelaan 25, 6229 HX Maastricht, The Netherlands; 3 Department of Surgery, Maastricht University Medical Center +, P. Debyelaan 25, 6229 HX Maastricht, The Netherlands; 4 Department of Plastic and Reconstructive Surgery, University of Texas Southwestern Medical Center, 1801 Inwood Road, Dallas, TX 75390 USA; 5 Division of Plastic Surgery, Houston Methodist Hospital, 6565 Fannin St, Houston, TX 77030 USA

**Keywords:** Breast reconstruction, Sensation, Quality of Life, Neurotisation, Nerve coaptation

## Abstract

**Purpose:**

The number of breast cancer survivors continues to grow. Due to refinements in operating techniques, autologous breast reconstruction has become part of standard care. Impaired sensation remains a debilitating side effect with a significant impact on the quality of life. Microsurgical nerve coaptation of a sensory nerve has the potential to improve sensation of the reconstructed breast. This study investigates the effect of improved sensation of the reconstructed breast on the quality of life in breast cancer survivors.

**Methods:**

A retrospective cohort study was performed in the Maastricht University Medical Center. Patients undergoing a DIEP flap breast reconstruction between January 2015 and January 2016 were included. The primary outcome was quality of life (BREAST-Q domain ‘physical well-being of the chest’). The Semmes–Weinstein monofilaments were used for objective sensation measurement of the reconstructed breast(s).

**Results:**

Eighteen patients with and 14 patients without nerve coaptation responded. Nipple reconstruction was the only characteristic that differed statistically significant between both groups (*p* = 0.04). The BREAST-Q score for the domain physical well-being of the chest was 77.89 ± 18.89 on average in patients with nerve coaptation and 66.21 ± 18.26 in patients without nerve coaptation (*p* = 0.09). Linear regression showed a statistically significant relation between objectively measured sensation and BREAST-Q score for the domain physical well-being of the chest with a regression coefficient of − 13.17 ± 3.61 (*p* < 0.01).

**Conclusions:**

Improved sensation in the autologous reconstructed breast, with the addition of microsurgical nerve coaptation, has a statistical significant positive impact on the quality of life in breast cancer survivors according to the BREAST-Q.

## Purpose

Although the incidence of breast cancer continues to grow, so do survival rates [1, 2]. Thus, the quality of life of breast cancer survivors has become of great importance. Although breast conservation therapy continues to be a major part of breast cancer treatment, mastectomy numbers continue to grow. In addition, more prophylactic (contralateral) mastectomies are being performed [3–6]. Approximately 40% of patients with invasive breast cancer and 33% of patients with ductal carcinoma in situ undergo mastectomy [7–9].

A mastectomy has a negative impact on body image, which in women is partially determined by a sense of femininity and attractiveness [10, 11]. Fortunately, breast reconstruction has shown to improve body image and quality of life [12–15]. The number of women undergoing breast reconstruction with either autologous tissue transfer or implants, is generally low and varies from 5 to 30% [16]. Preoperative referral to a plastic surgeon might have an influence on these numbers [17, 18]. Autologous reconstruction (41% of breast reconstructions) has shown superior results on quality of life if compared to implant reconstruction (61% of breast reconstructions) [19]. Due to refinements in operating techniques, autologous breast reconstruction has become part of standard breast cancer care.

Impaired sensation in the transposed skin and surrounding skin envelope after autologous breast reconstruction remains a debilitating side effect with a negative impact on the quality of life [20]. Sensation of the breast consists of different aspects: temperature, tactile, pain and erogenous sensation. Cases of burns or injuries of the insensitive autologous reconstructed breast have been described previously [21, 22], emphasizing the protective function of breast sensation and the importance of restoration of pain and temperature sensation in the reconstructed breast. Emotional reasons such as the need to feel feminine and sexually attractive again have been described in previous studies as decisive factors to opt for an autologous breast reconstruction. Furthermore, the question ‘does your reconstructed breast feel like your own?’, seems to be the most important determinant in patient satisfaction [23, 24]. It is questionable whether these goals can be achieved by reconstructing a mound of soft-tissue, although aesthetically pleasing, without any sensation. We believe restoring tactile sensation of the reconstructed breast enables women to experience their breast like ‘of their own’. This hypothesis is supported by a recent article in the New York Times that also emphasizes the importance of this topic to society [25]. This explains the recent trend to perform a microsurgical nerve coaptation of the sensory nerve of the DIEP flap to the 2nd or 3rd intercostal nerve. This addition to the technique has shown to improve sensation of the reconstructed breast [26–31].

In this study, the effect of sensory nerve coaptation in patients with a DIEP flap breast reconstruction on the quality of life as measured by the BREAST-Q when compared to standard reconstruction without nerve coaptation was evaluated for the first time.

## Methods

This study was conducted according to the STROBE guidelines and was approved by the medical ethical committee of the Maastricht University Medical Center [32].

### Patient population

We performed a retrospective cohort study in the Maastricht University Medical Center in Maastricht, the Netherlands. Participants included all consecutive patients, who underwent a DIEP flap breast reconstruction with or without nerve coaptation, between January 2015 and January 2016 in our center. Because of the retrospective design of this study, blinding patients with respect to sensory nerve was not possible. Medical records of 143 potential candidates were screened for a follow-up appointment at the Maastricht University Medical Center in January 2017 to obtain informed consent and to measure sensation of the reconstructed breast(s). The BREAST-Q and five additional questions, specifically about sensation in the reconstructed breast(s), with a self-addressed postage-paid return envelope were sent to consenting patients.

### Data collection

A chart review of all included participants was performed to compile data on the following patient characteristics: nerve coaptation, age, BMI, (neo) adjuvant chemo- and/or radiotherapy, reconstructive timing (immediate versus delayed), unilateral versus bilateral reconstruction, reason for mastectomy (prophylactic versus malignancy), complications, stage of reconstruction (nipple reconstruction already performed or not yet), and follow-up.

Patients were invited to complete the BREAST-Q questionnaire (Dutch for the Netherlands, reconstruction module) and five additional questions about breast sensation in particular (Table 1). If Dutch was their second language, an interpreter was suggested. The raw BREAST-Q scores were converted into domain scores using the QScore software programme (Memorial Sloan Kettering Cancer Institute, New York). The domain scores range from 0 to 100 per domain. A higher score indicates a better quality of life. Patients were asked to complete the domains that are likely to be influenced by sensation of the breast: physical well-being of the chest, psychosocial well-being, sexual well-being, satisfaction with breasts and satisfaction with outcome. These domains were used as separate outcome variables, as a total BREAST-Q score cannot be computed. The primary outcome was defined as the domain physical well-being of the chest, since we deemed it the most important quality of life measure related to sensation after breast reconstruction. The remaining domains and the five additional questions were considered as secondary outcomes. The five additional questions were scored on a 5-point Likert-type scale which was later dichotomized (Table 1).

**Table 1:** Additional questions concerning sensation of the reconstructed breast, scored on a 5-point Likert scale with dichotomizing process and codes under each question

1. Do you have sensation in your reconstructed breast(s)?				
Very much	Much	A bit	Little	Very little
Dichotomized: very much – a bit = 1, little – very little = 0				
2. Does the sensation in your reconstructed breast resemble the sensation of your healthy breast before operation?				
The same	Almost the same	Similar	A bit similar	Totally different
Dichotomized: the same – a bit similar = 1, totally different = 0				
3. How important do you find sensation in your breast?				
Very important	Important	Neutral	Not very important	Not important
Dichotomized: very important – important = 1, neutral – not important = 0				
4. Do you find it important that your reconstructed breast has sensation?				
Very important	Important	Neutral	Not very important	Not important
Dichotomized: very important – important = 1, neutral – not important = 0				
5. How important is the return of sensation in your reconstructed breast for your overall satisfaction with the result of the operation?				
Very important	Important	Neutral	Not very important	Not important
Dichotomized: very important – important = 1, neutral – not important = 0				

The Semmes–Weinstein monofilaments were used for sensation measurement of the reconstructed breast(s) [33]. The researcher was blinded for the treatment group of the patient (with or without nerve coaptation). Each monofilament value represents the logarithm of the force in milligrams required to bend the monofilament. Therefore, a thinner monofilament requires less pressure to bend and, if felt by the patient, represents improved one point static discrimination compared to a patient who is not able to feel it. Perpendicular pressure was applied to the same spot until monofilament bending was noted each time for a duration of 1.5 s, three times in succession, with intervals of 1.5 s. Testing started with the thinnest monofilament and progressed to monofilaments of increasing pressure until touch was identified in at least one out of three times by the patient. Patients were asked to lay on their back and close their eyes, measurements took place in a quiet room. The different sites were tested in a random sequence to ensure touch at a particular site could not be predicted [24].

**Fig. 1:**
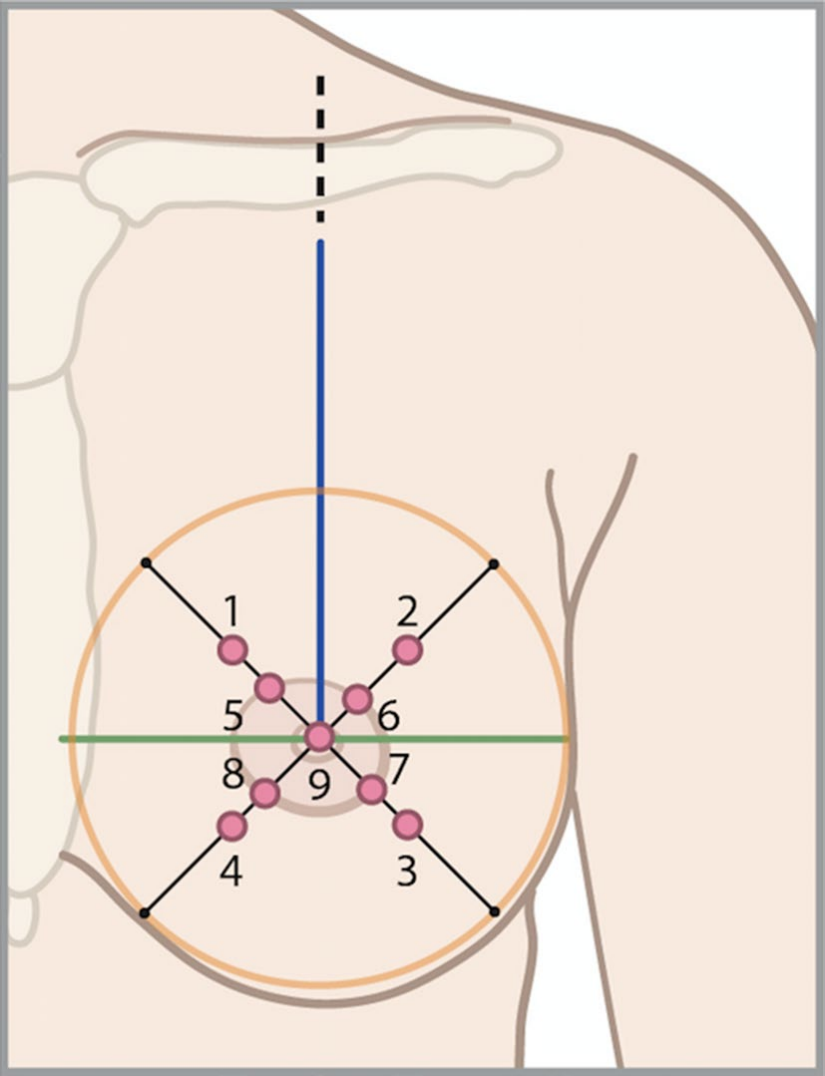
Anatomical landmarks of the breast for the nine sensation measurement points. The breasts were divided into four quadrants by two lines; line 1 (blue) was drawn from the mid of the clavicle to the nipple; line 2 (green) was drawn perpendicular on line 1. A circle (orange) was drawn around the breast tissue defined by the inframammary and supramammary crease. In each quadrant, a line (black) was drawn at 45°. On the middle of these four lines, sensation was measured. In addition, measurements were done on the areola in each quadrant

The areas to be measured were predefined by anatomical references; nine areas were tested in each breast. The breasts were divided into four quadrants by two lines; a vertical line was drawn from mid clavicle to the nipple; a horizontal line was drawn perpendicular to the first line at the nipple level. A circle was drawn around the breast tissue defined by the inframammary and supramammary crease which were determined by using the manoeuvre described by Marchac and de Olarte [34]. The breast was displaced cranially, caudally, medially, and laterally to define the respective borders of the breast. Thus, a circle with four quadrants was created with the nipple as it is centre. Each circle quadrant was bisected with a line drawn at 45°. Sensation was measured on the middle of each line and in the areola in each quadrant, and on the nipple (Fig. 1).

### Operating technique: nerve coaptation

A nerve coaptation of the 2nd or 3rd intercostal nerve to a sensory nerve of the DIEP flap was performed [35]. Nerve coaptation was performed under the microscope using two epineural stitches with 10–0 nylon. To fix the nerve, some tissue glue was added after coaptation was completed. On average, this extended the operation time by 15 min with no increased risk of complications. Neuropathic pain was not observed.

### Statistical analysis

As this was a pilot study, we did not compute the necessary sample size based on ample power to detect clinically relevant differences in BREAST-Q domain scores. Information on what differences can be regarded as clinically relevant, as well as measures of spread, were not available. Therefore, we included all records that were available at the time which fit the inclusion and exclusion criteria, to be able to estimate differences between the two groups as precisely as possible.

Baseline characteristics were compared between groups: DIEP-flap breast reconstruction with nerve coaptation (group 1) and DIEP-flap breast reconstruction without nerve coaptation (group 2). Continuous variables were reported as mean with standard deviation or median with range, depending on the distribution of the variables. Categorical variables were reported as absolute numbers and proportions. Continuous variables were compared using the independent *t* test or the Mann–Whitney *U* test, and categorical variables were assessed using χ^2^ test or Fisher’s exact test.

Sensation measurements of the breast(s) were averaged in order to create one value per patient. An independent samples *t* test was used to evaluate differences between both groups in mean monofilament value and mean BREAST-Q scores per domain. In addition, both simple and multiple (i.e. unadjusted and adjusted) linear regressions were used to estimate the crude and adjusted associations between objectively measured sensation and the BREAST-Q score domains. The adjusted regression coefficient was corrected for baseline characteristics that differed statistically significant between groups.

The five additional questions were analysed using both simple and multiple logistic regressions and were reported as odds ratios (OR) with a 95% confidence interval (CI).

A *p* value < 0.05 was considered to be statistically significant. All analyses were performed using IBM SPSS (Released 2013. Version 24.0. Armonk, NY: IBM Corp).

## Results

### Response rate

**Fig. 2:**
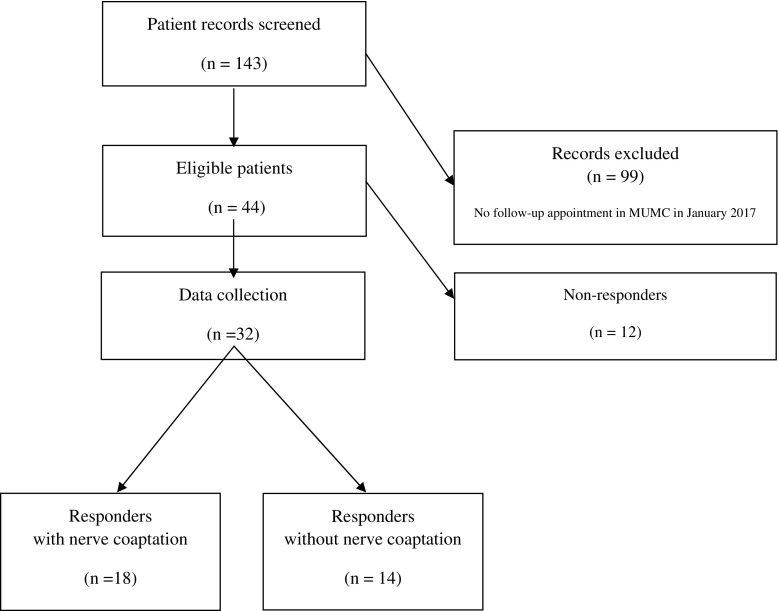
Patient enrolment flow chart

In total, 44 patients of 143 screened records were eligible. Eighteen patients with and 14 patients without nerve coaptation responded. This resulted in an overall response rate of 73% (32 out of 44). (Fig 2).

### Patient characteristics

Patient characteristics between the group of patients with and the group of patients without nerve coaptation were compared (Table 2). The variables age, BMI, chemotherapy, radiotherapy, reconstructive timing (immediate versus delayed), unilateral versus bilateral breast reconstruction, reason for mastectomy (prophylactic versus malignancy), and complications did not differ significantly between groups. However, stage of reconstruction (nipple reconstruction already performed or not yet) differed significantly between these two groups (*p* = 0.04). In the group of patients without nerve coaptation, 92.9% already underwent a nipple reconstruction compared to 44.4% of patients in the group of patients with nerve coaptation.

**Table 2:** Demographic characteristics of included patients

Variable	Nerve coaptation (*n* = 18)	No nerve coaptation (*n* = 14)	*p* value (2-sided)
Age at operation, mean in years ± SD	47.11 ± 9.2	47.71 ± 4.73	0.825
BMI, mean in kg/m^2^ ± SD	26.44 ± 2.77	28.21 ± 3.04	0.096
Chemotherapy, %			
Previous chemotherapy	44.4	57.1	0.476
No previous chemotherapy	55.6	44.9	
Radiotherapy, %			
Previous radiotherapy	22.2	14.3	0.672
No previous radiotherapy	77.8	85.7	
Reconstructive timing, %			
Immediate	61.1	42.9	0.305
Delayed	38.9	57.1	
Unilateral versus bilateral, %			
Unilateral	27.8	42.9	0.465
Bilateral	72.2	57.1	
Reason mastectomy, %			
Malignancy	66.7	42.9	0.178
Prophylactic	33.3	57.1	
Complication, %	33.3	14.3	0.412
Stage of reconstruction, %			
Nipple reconstruction	44.4	92.9	0.04*
No nipple reconstruction	55.6	7.1	
Follow-up in months ± SD	16.13 ± 3.24	14.76 ± 4.30	0.33

*Statistical significant results

### Sensory and quality of life results

The mean monofilament value in patients who underwent a breast reconstruction with nerve coaptation was lower 4.35 than in patients without nerve coaptation 5.30, which indicates better sensation in patients with nerve coaptation. Despite our small sample size, this mean difference was statistically significant *p* < 0.01. (Table 3) The mean score of the BREAST-Q domain physical well-being of the chest was also compared between both groups: the mean score of patients with nerve coaptation was higher (77.89) than without nerve coaptation (66.21) (*p* = 0.09), which suggests a higher quality of life in patients with nerve coaptation. (Table 3).

**Table 3:** Mean monofilament value and BREAST-Q score compared between patients with and without nerve coaptation

	Nerve coaptation(*n* = 18)	No nerve coaptation(*n* = 14)	*p* value (2-sided)
Monofilament value	4.35 [4.00–4.69]	5.30 [4.94–5.66]	< 0.01
Physical well-being chest	77.89 [68.51–87.27]	66.21 [55.67–76.76]	0.09

**Fig. 3:**
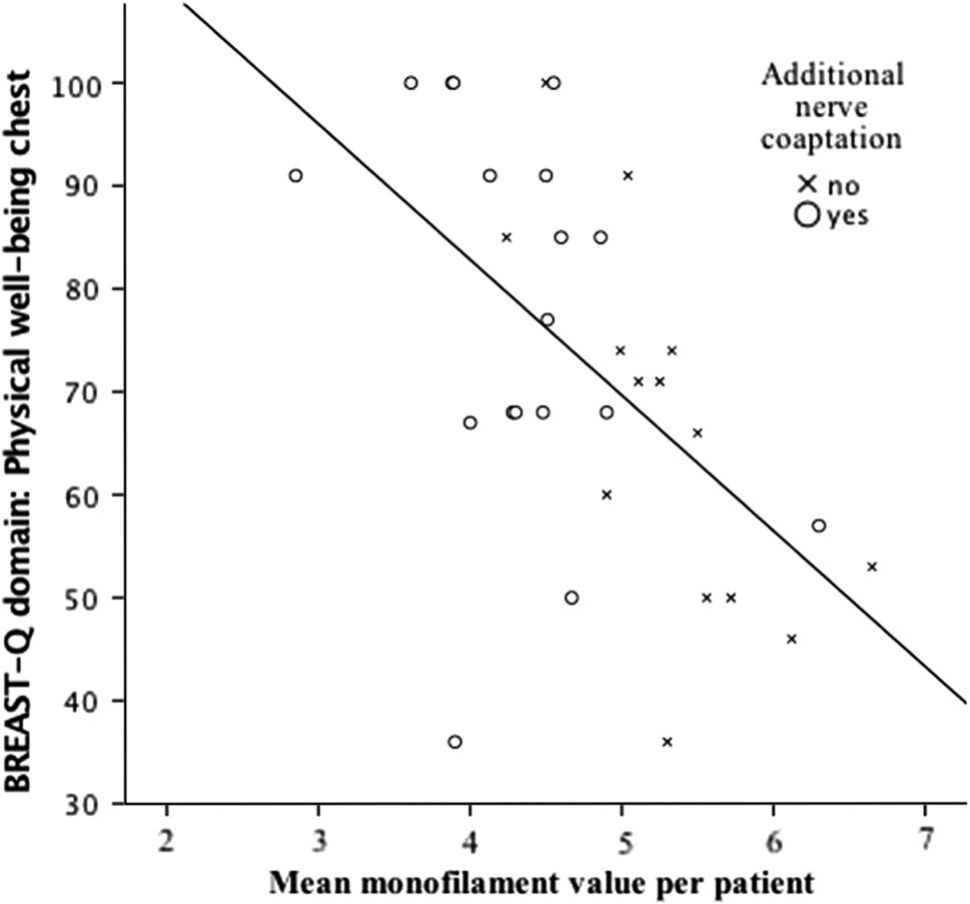
Linear regression: Relationship between quality of life and sensation in DIEP reconstructed breast. *y*-axis represents the value of the BREAST-Q domain physical well-being of the chest, values range from 1 to 100: a higher value represents a higher patient satisfaction. *x*-axis represents a mean monofilament value, which was calculated per patient, if a patient underwent a reconstruction bilaterally one mean value for both breasts was calculated. A scatterplot in which the circles represent the patients with nerve coaptation and the crosses represent patients without nerve coaptation is shown. The black line is the linear regression with a regression coefficient of − 13.17 ± 3.61 and a *R* ^2^ of 0.31 ± 16.26 (*p* < 0.01)

The relation between objectively measured sensation and BREAST-Q score for the domain physical well-being of the chest was explored in a linear regression model and showed a statistically significant correlation (*p* < 0.01) with a crude regression coefficient of − 13.17 and a *R* ^2^ of 0.31 (Fig. 3; Table 4). The adjusted regression coefficients were − 11.47 ± 2.53 for the variable monofilament with a *R* ^2^ of 0.40 (*p* < 0.01). (Table 4) The results of the crude and adjusted regression coefficients of the secondary outcome domains of the BREAST-Q of this study are shown in Table 4.

**Table. 4:** Crude and adjusted linear regression coefficients of primary and secondary outcome domains of the BREAST-Q in relationship to objectively measured sensation (mean monofilament value)

Independent	Dependent	Crude			Adjusted^a^		
		Coeff	CI	*p* value	Coeff	CI	*p* value (2-sided)
Physical well-being chest	Mean monofilament value	− 13.17	− 20.53–− 5.81	< 0.01	− 11.47	− 18.68–− 4.26	< 0.01
Satisfaction with breast	Mean monofilament value	− 2.20	− 10.62–4.79	0.45	− 2.33	− 10.34–5.70	0.56
Satisfaction with outcome	Mean monofilament value	− 8.02	− 17.74–1.71	0.10	− 6.89	− 16.73–2.95	0.16
Psychosocial well-being	Mean monofilament value	0.77	− 8.52–10.06	0.17	1.19	− 8.52–10.90	0.80
Sexual well-being	Mean monofilament value	0.66	− 10.07–11.39	0.90	2.29	− 8.50–13.08	0.67

*coeff* regression coefficient, *CI* confidence interval

^a^Adjusted for stage of reconstruction (nipple reconstruction already performed or not yet)

The relation between objectively measured sensation and BREAST-Q score for the domain physical well-being of the chest was also explored for primary and secondary breast reconstruction separately. Regression coefficient, *R* ^2^ and *p* value are, respectively, given for primary breast reconstruction: − 14.72, 0.27 and 0.03, and for secondary breast reconstruction: − 14.07, 0.42 and < 0.01.

A negative association of the monofilament value with the additional question 1 ‘*Do you have sensation in your reconstructed breast(s)?*’ was observed (OR 0.24, 95% CI 0.06–0.89). This suggests that a higher monofilament value, which represents less sensation, is statistically significant associated with answering negatively on this question.

A negative association of monofilament value with additional question 2 ‘*Does the sensation in your reconstructed breast resemble the sensation of your healthy breast before operation?*’ was also observed (OR 0.10, 95% CI 0.02–0.54). This indicates a higher monofilament value to be statistically significant associated with a negative answer on this question.

The remaining additional questions showed the following results: 64.52% of the respondents indicated to find breast sensation important to very important, 58.06% indicated that finding sensation in the reconstructed breast important was very important and 38.71% indicated that return of sensation in the reconstructed breast was important to very important for their overall satisfaction with the operation.

## Discussion

Breast reconstruction techniques have evolved greatly over the past decade, from focussing on survival of the transplanted tissue to minimizing morbidity and improving aesthetical outcome. Despite the efforts to recreate a natural looking breast, restoring sensation has been rather disregarded. Yet, we strongly believe that increased sensation of the reconstructed breast improves quality of life in breast cancer survivors. A study by Temple et al. in 2009 confirmed that innervation of the free TRAM flap breast reconstruction improves quality of life [20]. However, Temple et al. used general quality of life questionnaires to detect differences in quality of life thanks to improved breast sensation: the Medical Outcomes Study 36-Item Short Form, the Body Image after Breast Cancer Questionnaire and the Functional Assessment of Cancer Therapy–Breast quality-of-life. Validity of these instruments can be questioned. These instruments might be too general to evaluate quality of life related to breast sensation. As was suggested by Alderman and Chung in a discussion on the article by Temple et al., it is possible that factors other than breast sensation have influenced the results [36]. In this study, a more specific questionnaire (BREAST-Q) was used that was validated specifically among patients who underwent a mastectomy and a DIEP-flap breast reconstruction.

Our data showed a statistical significant improvement in objectively measured sensation of the reconstructed breast in the group of patients with nerve coaptation when compared to the group of patients without nerve coaptation. This is supported by many other studies [26–31]. However, an improvement in objectively measured sensation, like pressure sensitivity, does not unequivocally entail that the reconstructed breast feels more like a natural breast. The qualitative aspect of sensation is ignored if only objective sensation measurements are used; therefore, patient-reported outcomes are crucial. This is especially true with the rise in breast cancer survivors and the corresponding increased focus on patients’ quality of life as suggested by Wommack and Spiegel [37]. Moreover, the current health care economic environment mandates patient-reported outcomes and health-related quality of life research to justify added expense of increasingly complex surgical procedures.

This is the first study that measures quality of life using a questionnaire, which is specifically validated in patients who underwent a DIEP flap breast reconstruction (BREAST-Q), to evaluate the influence of breast sensation on the quality of life [38]. The domain physical well-being of the chest was chosen as primary outcome in this study since improved breast sensation will most likely influence this domain. The primary outcome of the current study, BREAST-Q domain physical well-being of the chest, was compared between patients with and without nerve coaptation. On average, the BREAST-Q score for the domain physical well-being of the chest was 11.68 points higher for the group with nerve coaptation. The smallest clinically significant difference of the BREAST-Q score is not yet defined. A study of the subjective significance to patients of changes in quality of life scores by the developers of the BREAST-Q, Pusic et al., suggests that a mean difference of 5–10 on the multi-item scales is perceived as a small clinical difference, 10–20 as a moderate and greater than 20 as a clinically important difference. Therefore, this difference might be considered moderately clinically relevant; however, we observed no statistically significant differences. The relatively small sample size of this pilot study is the likely cause of insufficient statistical power to detect clinically meaningful differences of this magnitude.

An additional linear regression model comparing objectively measured sensation with BREAST-Q score for the domain physical well-being of the chest was performed. This showed a highly statistically significant association between improved sensation and increased BREAST-Q score for the domain physical well-being of the chest, even if we corrected for statistical significant differences in baseline characteristics. Stage of reconstruction (nipple reconstruction already performed or not yet) was the only statistical significant baseline characteristic between both groups, which differed in favour of the group without nerve coaptation. Therefore, the positive effect on quality of life, thanks to additional nerve coaptation, might be rather underestimated.

This linear regression model was also studied for primary and secondary breast reconstructions separately because the surface of the transposed skin is usually much bigger in secondary breast reconstructions. Therefore, we expected the effect to be higher in patients who underwent a secondary breast reconstruction. This hypothesis might be confirmed by our analysis: a larger proportion of difference in BREAST-Q score can be explained by objective improvement of sensation for secondary breast reconstruction (42%) if compared to primary breast reconstruction (27%) and the statistical significance was more profound for secondary (*p* = 0.009) than for primary (*p* = 0.032) breast reconstruction.

Statistically significant regression coefficients of all the above models showed that a perceptible difference in the quality of life of breast cancer survivors can be explained by improved sensation of the DIEP flap reconstructed breast. This suggests that focussing on improved sensation of the reconstructed breast is worth the effort. A note of caution is due here since purposely blinding of patients could not be carried out, due to the retrospective design of this study. This is an important issue for future research. Further studies, which take this possible bias into account, will need to be undertaken, e.g. a larger randomized double-blinded prospective study would provide more data on this issue.

The BREAST-Q domain physical well-being of the chest contains 16 items and only eight items explore sensation of the reconstructed breast. Of those, only one question investigates the effect of positive sensation in the reconstructed breast. Additionally, the domain satisfaction with breasts contains two questions on breast sensation; however, this domain contains 25 items. We believe the domain of physical well-being of the chest to be more specific for breast sensation. Unfortunately, BREAST-Q questions were not validated to be analysed separately.

Even though the BREAST-Q (reconstruction module) is currently the most specific validated instrument to measure quality of life in breast cancer patients who underwent an autologous breast reconstruction, it is still not developed to measure differences in quality of life specifically related to improved sensation. Development and validation of a specific questionnaire could be of great value. Previous publications showed the question ‘does your reconstructed breast feel like your own?’, to be the most important determinant in patient satisfaction [23, 24]. However, these studies did not use validated questionnaires. In the current study, the group of patients with nerve coaptation were statistically more likely to answer positively on the question ‘Does the sensation in your reconstructed breast resemble the sensation of your healthy breast before operation?’. This might indicate a higher patient satisfaction. Furthermore, 64.52% of the respondents indicated to find breast sensation important to very important.

In this study, five patients who received a bilateral DIEP-flap breast reconstruction with only unilateral nerve coaptation, due to anatomical variations or technical difficulties, were excluded from the analysis, because BREAST-Q scores are analysed per patients rather than per flap. However, these patients can perfectly compare the results of a DIEP-flap breast reconstruction with and without nerve coaptation. Telephone interviews with these five patients taught us that these women experienced the reconstructed breast with nerve coaptation to feel less numb. Also, the reconstructed breast with nerve coaptation felt more like their own. This again shows the importance of sensation of the reconstructed breast; however, we are aware of the fact that these are empirical observations.

## Conclusions

This pilot study suggests that improved sensation in the autologous reconstructed breast, via an additional microsurgical nerve coaptation, has a statistical significant positive impact on the quality of life in breast cancer survivors according to the BREAST-Q. Therefore, nerve coaptation has a direct impact on patient outcomes and should be considered a useful addition to the surgical technique of autologous breast reconstruction.
